# Mr. Crowfoot, in Explanation

**Published:** 1802-02

**Authors:** W. Crowfoot

**Affiliations:** Beccles


### *To the Editors of the Medical and Phyfical Journal.*

Gentlemen,

I Shall think myfelf much obliged, if you will have the goodnefs to explain to your readers, why you called my card or addrefs to the Editors, *a private letter*; becaufe, a partial extract of it only being printed, and that placed in the preface to your criticifm upon my pamphlet, it conveys an idea that it was written to deprecate your cenfure, which, in truth and juftice both to yourfelves and my own character, I muft wifh to obviate.*

I feel the more anxious to remove all poffibility of miftake, as with inattentive or carelefs readers, it appears I have already fuffered

---

* It did not occur to us, that any one of our readers could draw fuch an inference as this from our ufe of the words *private letter*. There is no occafion to deprecate the cenfure of critics who give their names to the public. The only feverity they can exercife, is, to fay nothing when they find nothing to commend. Edit.

fuffered from mifconception, and its effects, mifreprefentation; not to fpeak of the uncourteous or malevolent commentator, who may look for gratification in perplexing a queftion which he cannot anfwer. Mr. H. Davies, in your laft number, page 74, &c. has related two *fatal* cafes of Apoplexy arifing from extravafation, and then fubjoins, "he fhould have judged the exhibition of an *emetic* an injudicious, if not a dangerous remedy;" meaning, I prefume, that the writer of a *certain case of apoplexy*† would have adminiftered fuch a medicine.

In this remark, it is evident your correfpondent confounds the cafe of nervous apoplexy, in which emetics are frequently advifable, with cafes arifing from plethora, wherein probably no man would think to employ them. And as the firft patient of Mr. Davies muft have been in a ftate (as often happens in fuch fatal attacks) to render it *impossible* to give an emetic, I think the obfervation might very well have been fpared.

If Mr. D. condefcends to read my pamphlet, he will perceive, and I have no doubt candidly acknowledge, the injuftice done to my opinions. I am, &c.

† As the phyfician, Dr. L———w determined the cafe to be *real apoplexy*, though *his patient* was free from every fymptom which denotes extravafation, exudation, or effufion; it remained for me to prove, and I truft it has been done fatisfactorily, that if this patient's cafe was apoplexy, which I will not difpute, it was unqueftionably the nervous apoplexy of Dr. Kirkland.

